# A mutation in the NADH-dehydrogenase subunit 2 suppresses fibroblast aging

**DOI:** 10.18632/oncotarget.3298

**Published:** 2015-03-24

**Authors:** Marianne Schauer, Tina Kottek, Madeleine Schönherr, Animesh Bhattacharya, Saleh M Ibrahim, Misa Hirose, Rüdiger Köhling, Georg Fuellen, Ulf Schmitz, Manfred Kunz

**Affiliations:** ^1^ Department of Dermatology, Venereology and Allergology, University of Leipzig, Leipzig 04103, Germany; ^2^ Department of Dermatology, Allergology and Venereology, University of Lübeck, Lübeck 23538, Germany; ^3^ Department of Physiology, University of Rostock, Rostock 18057, Germany; ^4^ Department of Biostatistics and Informatics in Medicine and Ageing Research, University of Rostock, Rostock 18057, Germany; ^5^ Department of Systems Biology and Bioinformatics, University of Rostock, Rostock 18057, Germany

**Keywords:** aging, senescence, mitochondria, skin fibroblasts, p38MAPK signalling

## Abstract

Mutations of mitochondrial (mt)DNA cause a variety of human diseases and are implicated in premature aging syndromes. Here we investigated a single nucleotide exchange (leucine to methionine) at position nt4738 in the mitochondrial NADH dehydrogenase subunit 2 (Nd2) gene of the respiratory chain. Primary fibroblasts derived from the conplastic mouse strain C57BL/6J-mtALR/LTJ with mutant enzyme, possessed high enzyme activity and ATP production and low ROS production. Furthermore, Nd2-mutant fibroblasts expressed lower senescence markers. Transcriptome analysis revealed that the members of the p38MAPK pathway were significantly downregulated in Nd2-mutant mice. In agreement, inhibition of p38MAPK with SB203580 enhanced proliferation and reduced cytokine secretion in fibroblasts. In Nd2-mutant mouse skin, the amount of Ki67-positive cells was significantly higher than in control skin. The higher amount of Ki67-positive cells and the thicker epidermis in Nd2-mutant mice strongly supported the *in vitro* data. In conclusion, Nd2 is a mitochondrial gene, involved in age-related signaling pathways.

## INTRODUCTION

NADH dehydrogenase is the first enzyme complex of the mitochondrial respiratory chain (complex I) and oxidizes NADH to liberate electrons to support the translocation of protons across the inner membrane to create a proton gradient [1]. The mammalian complex I has a molecular size of 1 MDa and contains 45 subunits, 7 of which are encoded by mitochondrial DNA and 38 by nuclear DNA [2]. The seven mitochondrial-encoded subunits (ND1–6 und ND4L) play a significant role in diverse pathological processes. The NADH dehydrogenase subunit 2 enzyme is encoded by the *ND2* gene and has a size of 39 kDa. The function of ND2 is not fully understood. A mutation in the *ND2* gene (T4681C) was found in patients with Leigh Syndrome, a neurodegenerative disease characterized by bilateral symmetric lesions in basal ganglia and subcortical brain regions [3]. It is postulated that ND2 is involved in proton translocation across the inner mitochondrial membrane, thereby contributing to the pH regulation of the cell. The hydrophobic subunit of complex I is highly conserved throughout evolution from bacteria to mice to man, arguing for a central physiological role [4]. Specific mitochondrial point mutations cause the development of different diseases and are involved in the processes of aging. For example, Alzheimer's disease is associated with dysfunction in the mitochondrial electron transport enzyme cytochrome c oxidase [5, 6]. Genetic alterations in mitochondrial DNA may cause a decline in mitochondrial oxidative phosphorylation, increased mitochondrial production of reactive oxygen species and an enhanced amount of oxidative damage to DNA, proteins and lipids [5]. A direct link between mtDNA and mammalian aging was shown in mice with a proofreading-deficient-, catalytic subunit of mitochondrial DNA polymerase γ (PolgA). In these mice, the accumulation of mtDNA mutations was linked to an impaired respiratory chain function and increased cellular apoptosis. The mutated mice had a shorter life span and developed an age-related phenotype with hair loss, osteoporosis and kyphosis [7].

Cellular senescence is a contributor to organismal aging. Senescent cells have been detected *in vivo* in different tissues in mice and humans [8]. Senescence can be triggered by various extrinsic and intrinsic factors, and was first observed in cell culture [9]. In superoxide dismutase 2 (SOD2)-deficient mice was shown that natural aging is linked by decreased mitochondrial activity in complex II and increased cellular senescence in mouse skin. Accompanied by the idea that these events are linked, constitutive SOD2-deficency resulted in mitochondrial dysfunction and cellular senescence in the epidermis of the skin [10]. For *in vitro* or *in vivo* characterization of senescent cells show reduced proliferation, enhanced β-galactosidase activity, expression of senescent-associated heterochromatin foci (SAHF) or senescence-associated DNA damage foci (SDF), activation of different stress pathways such as p53 or p38MAPK, and development of a senescence-associated secretory phenotype (SASP) [11].

Comparing Japanese centenarians with younger control individuals shows an association of the *ND2* Leu273Met polymorphism (A5178T) with longevity [12, 13]. Additionally, individuals with this polymorphism provide resistance against ROS-mediated diseases like myocardial infarction, atherosclerosis and high blood pressure [4, 14]. Mice with the corresponding polymorphism of a leucine to methionine transition at position nt4738 (known as the alloxan-resistant (ALR) mouse strain) showed resistance to the development of spontaneous type-I diabetes and had reduced ROS production compared with NOD (non-obese diabetic) mice [14].

The present study demonstrates that an *Nd2* mutation at position nt4738 in the conplastic mouse strain C57BL/6J-mt^ALR/LtJ^ suppresses ROS-induced-senescence in primary skin fibroblasts. Microarray analysis suggests that downregulation of the p38MAPK pathway is a major contributor to reduced senescence induction. Inhibition of p38MAPK (with inhibitor SB203580) indeed led to a stronger reversion of the expression of senescence markers in the *Nd2*-mutant strain compared with control mice. Taken together, our data show that the *Nd2* mutation is protective for fibroblasts against ROS-induced cellular senescence, which may also have an impact on organismal aging.

## RESULTS

### Skin fibroblasts of *Nd2*-mutant mice show significantly reduced ROS levels

**Figure 1:**
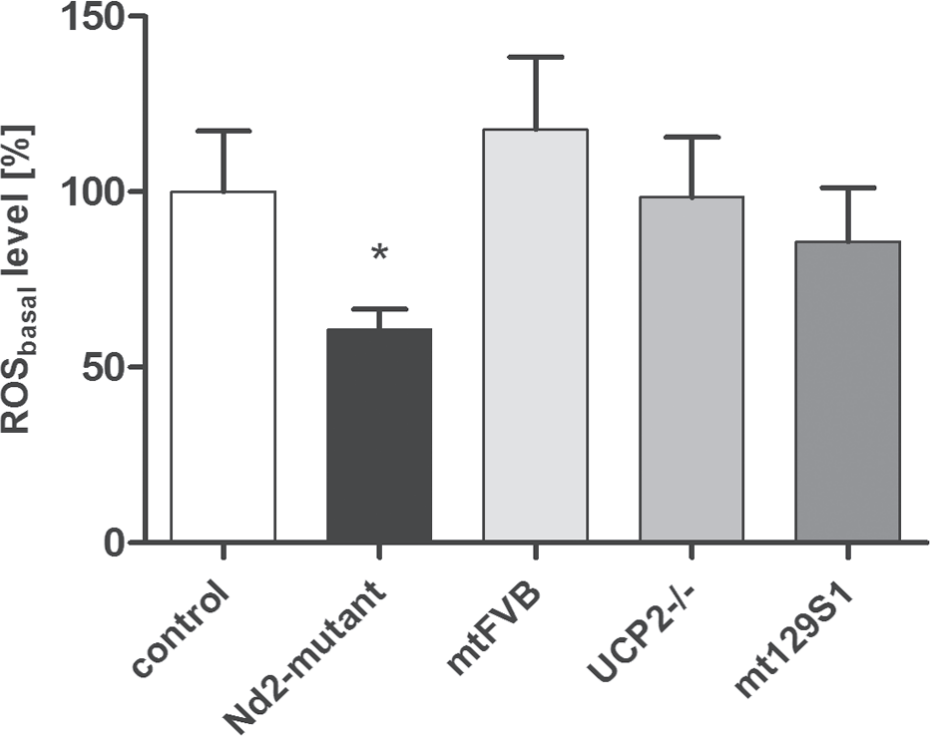
ROS levels of four different conplastic mouse strains and uncoupling protein 2-knockout strain Fibroblasts were isolated from skin of 12-month-old mice. Intracellular basal ROS levels were determined from isolated primary fibroblasts using DCFH-DA method. Data are given as mean ± SEM of 4–12 independent experiments; *p < 0.05. Control: C57BL/6J-mt^AKR/J^; *Nd2* mutant: C57BL/6J-mt^ALR/LtJ^; mtFVB: C57BL/6J-mt^FVB/NJ^; UCP2−/−: C57BL/6J-UCP2−/−; mt129S1: C57BL/6J-mt^129S1/SvlmJ^.

The mitochondrial production of reactive oxygen species (ROS) is physiological but has also been described in ROS-induced aging and senescence. In the present study, the question was addressed whether mitochondrial mutations in respiratory chain genes influence the cellular senescence of skin fibroblasts and skin aging. For this purpose, four conplastic mouse strains were used with identical nuclear DNA but with different mutations in the mitochondrial genome and uncoupling protein 2-knockout strain [15]. C57BL/6J-mt^AKR/J^ mice were used as reference strain; these differ only in the respective position of the mitochondrial genome from the experimental strains (Table S1). First, the basal ROS production of primary skin fibroblasts of aged mice (12-month-old) of different conplastic strains and uncoupling protein 2-knockout strain was tested. The fibroblasts of strains C57BL/6J-UCP2−/−, C57BL/6J-mt^FVB/NJ^ and C57BL/6J-mt^129S1/SvlmJ^ showed no significant difference in basal ROS production as compared with the control strain (Figure 1). However, the C57BL/6J-mt^ALR/LtJ^ strain with the mutation in NADH dehydrogenase subunit 2 (*Nd2*; position nt4738) showed significantly reduced basal ROS levels. Based on this data, the *Nd2*-mutant mouse strain (C57BL/6J-mt^ALR/LtJ^) was chosen to analyze the impact of a specific point mutation on fibroblast senescence and skin aging.

### Skin fibroblasts of *Nd2*-mutant mice show reduced expression of cellular senescence markers

**Figure 2:**
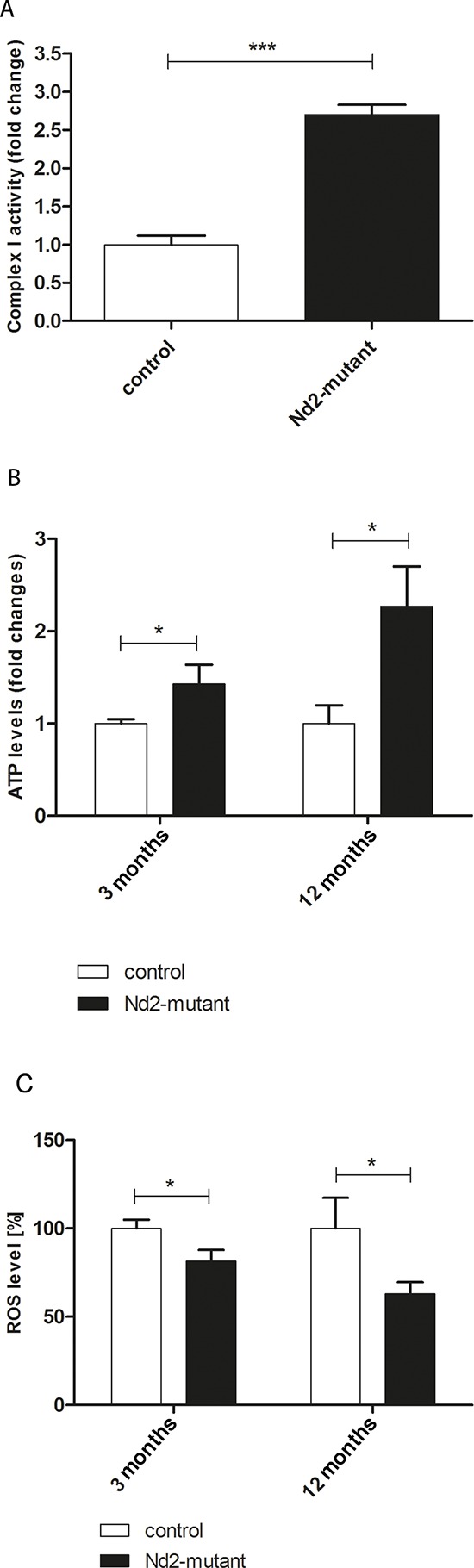
Baseline characteristics of mitochondrial capacity of dermal fibroblasts of conplastic mouse strains **A.** Comparison of enzymatic activity of complex I in control and *Nd2*-mutant mice. The assay was performed in three independent experiments with isolated mitochondria of three different 12-month-old mice. ***p < 0.001. **B.** Comparisons of ATP levels in younger (3-month-old) and older (12-month-old) control and *Nd2*-mutant mouse fibroblasts. *n* = 5–9, *p < 0.05. **C.** ROS level determination of isolated fibroblasts of 3-month- and 12-month-old control and *Nd2*-mutant mice. *n* = 5–11, *p < 0.05. Data are given as mean ± SEM.

Next the enzymatic activity of complex I in *Nd2*-mutant and control primary skin fibroblasts was investigated. Complex I is of central importance for ATP production, and the *Nd2* mutation could thus result in either enhanced or reduced enzyme activity and energy production. Complex I activity was determined by measurement of the oxidation of NADH to NAD^+^ from isolated mitochondria of *Nd2*-mutant and control fibroblasts. Fibroblast mitochondria of the *Nd2*-mutant strain showed a 2.6-fold higher complex I activity as compared with control mitochondria (Figure 2A). Thus, the *Nd2*-mutant mice may have an enhanced *Nd2* baseline activity. We also tested if the enzyme activity correlated with protein expression of complex I in control and *Nd2*-mutant mice. The basal and the stress-induced protein expression of *Nd2* did not differ in fibroblasts between both strains (Figure S1), however.

To further characterize the mitochondrial function of skin fibroblasts, ATP production was measured in fibroblasts of young (3-month-old) and aged (12-month-old) mice. *Nd2*-mutant skin fibroblasts produced significantly higher amounts ATP at both time points than control fibroblasts (Figure 2B). As shown above, the ROS levels in *Nd2*-mutant fibroblasts were significantly lower than in control fibroblasts, as determined by the DCFH-DA method (Figure 1, Figure 2C). These findings suggest that higher complex I enzyme activity leads to higher ATP production and decreased ROS production, and we hypothesize that the induction of senescence in skin fibroblasts of *Nd2*-mutant mice may thus be reduced during the aging process.

**Figure 3:**
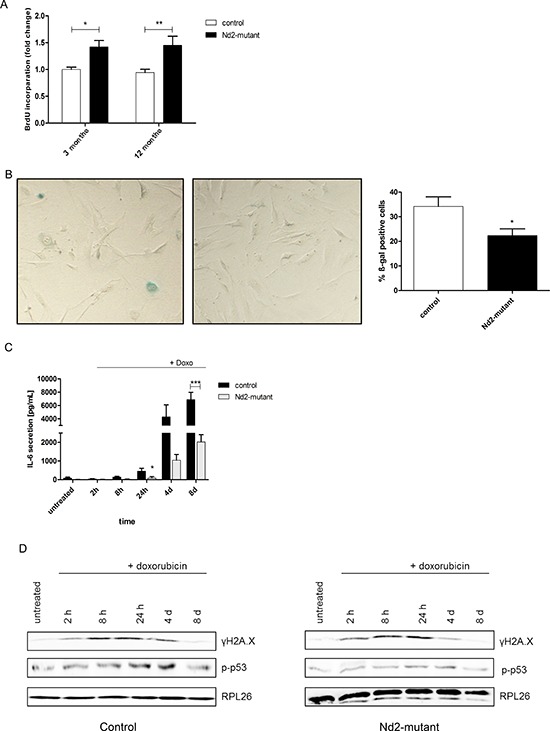
Characteristic features of cellular senescence in skin fibroblasts of *Nd2*-mutant mouse strain **A.** For primary skin fibroblasts of 3-month-old and 12-month-old mice, cell proliferation of *Nd2*-mutant or control mouse fibroblasts was analysed by BrdU incorporation assay. *n* = 3–13, *p < 0.05, **p < 0.01. **B.** Fibroblasts from 12-month-old mice were fixed and stained for SA-ß-gal 24 h after seeding. Representative picture of SA-β-gal-staining is shown (left panel). The number of positive blue cells was divided by the total number of counted cells resulting in the percentage of ß-gal-positive cells (right panel). *n* = 3, *p < 0.05. Data are expressed as mean ± SEM. **C.** Fibroblasts of 12-month-old *Nd2*-mutant and control mice were treated for 1 h with 250 nM doxorubicin and supernatants were collected and analysed for IL-6 concentrations by ELISA at indicated time points. *n* = 6–7, *p < 0.05 ***p < 0.001. Data expressed as mean ± SEM. **D.** Fibroblasts of 12-month-old *Nd2*-mutant and control mice were treated for 1 h with 250 nM doxorubicin and whole cell lysates were collected at the indicated days thereafter. Protein expression of γH2A.X and p-p53 was analysed by immunoblotting. Lysates were pooled from fibroblasts of three different mice. RPL26 was used as loading control.

Therefore, primary skin fibroblasts isolated from 3- and 12-month-old *Nd2*-mutant mice and control mice were analyzed for the expression of markers of cellular senescence. At a cellular level, senescence in primary fibroblasts might be detrimental for skin function in general. In a first series of experiments, the impact of the *Nd2* mutation on cellular proliferation of primary skin fibroblasts in both strains was analyzed. For this purpose, BrdU incorporation assays were performed. *Nd2*-mutant fibroblasts showed a more than 50% higher proliferation rate than control fibroblasts in both 3- and in 12-month-old mice (Figure 3A).

In subsequent analyses, we examined the senescence-associated β-galactosidase (SA-β-gal) activity, a common marker of cellular senescence, in fibroblasts isolated from 12-month-old mice of both strains. SA-β-gal activity in skin fibroblasts of the *Nd2*-mutant strain was significantly lower than that of control fibroblasts (Figure 3B). This suggests that *Nd2*-mutant mouse fibroblasts have a better resistance to induction of cellular senescence as compared with fibroblasts of the control strain.

A further characteristic feature of senescent cells is the development of a senescence-associated secretory phenotype (SASP). Senescent cells increase the secretion of various cytokines, chemokines and matrix metalloproteinases, which alter the local tissue environment [16]. Here we measured a prominent component of the SASP, IL-6. To induce a SASP by genotoxic stress, cells were exposed to doxorubicin, and cytokine secretion was measured at different time points. Doxorubicin is a well-known antitumor anthracycline antibiotic, which induces genotoxic and oxidative stress and cellular senescence in different cell types [17, 18]. After treatment of fibroblasts with low dose (250 nM) doxorubicin, an increase in IL-6 secretion was observed in both mouse strains over 8 days. Interestingly, *Nd2*-mutant fibroblasts showed a significantly lower IL-6 secretion compared with control fibroblasts (Figure 3C).

Cellular senescence is further characterized by an increased expression of a number of DNA damage-associated proteins and followed by activation of the p53 pathway. Thus, the activation and expression of γH2A.X and p-p53 protein was examined by immunoblot analysis (Figure 3D). Whole cell lysates were prepared from fibroblasts at different time points after doxorubicin treatment until cells developed a senescent phenotype (0 – 8 days). First, the expression of γH2A.X as a typical marker of DNA damage and cellular senescence was analyzed [19]. Both strains showed activation and phosphorylation at serine 139 of H2A.X, indicated as γH2A.X. γH2A.X expression increased 2 h after doxorubicin treatment in fibroblasts of *Nd2*-mutant and control mice. The activation of γH2A.X remained stable until 24 h in *Nd2*-mutant fibroblasts, but in control fibroblasts until 4 d. Interestingly, doxorubicin-induced DNA damage led to a similar phosphorylation kinetic of p53 in *Nd2*-mutant and control fibroblasts. In both strains, p53 showed increased phosphorylation until 24 h. At 4d, the p-p53 expression started to decrease in both strains. The p-p53 reaction pattern looked similar in both strains; however in Nd2 mutant strain fibroblasts overall p-p53 expression was weaker.

**Figure 4:**
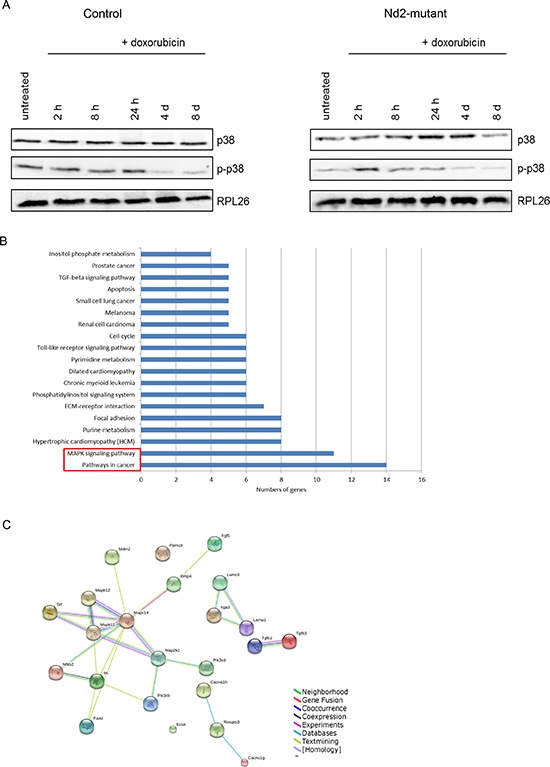
Transcriptome analysis of fibroblasts from *Nd2*-mutant and control mice microarray **A.** Immunoblotting of total p38MAPK and p-p38MAPK of fibroblasts from 12-month-old *Nd2*-mutant and control mice. Lysates were pooled from fibroblasts of three different mice. RPL26 was used as loading control. **B.** Total RNA was extracted from fibroblasts of *Nd2*-mutant and control mice and subjected to transcriptome analysis using MouseRef-8 v2.0 Expression BeadChips. Significantly enriched downregulated genes were grouped by pathways using DAVID Bioinformatics Resources 6.7. **C.** Map of genes interactions downregulated in “pathways in cancer” and “MAPK signaling pathway” generated by STRING 9.1 analysis.

Next, p38MAPK expression and phosphorylation was tested (Figure 4A). Diverse senescence stimuli activate this kinase, which in turn regulates the senescence-associated secretory phenotype by increasing NF-κB activity independent of other senescence pathways [16]. After doxorubicin treatment, total p38MAPK (p38α, β, γ) expression increased until 4 d in mutant mouse fibroblasts. In control fibroblasts, total p38MAPK expression was constantly expressed. p38MAPK is activated by phosphorylation at threonine 180 and tyrosine 182. In *Nd2*-mutant fibroblasts, p38MAPK was transiently phosphorylated at 2 h and phosphorylation decreased thereafter. In contrast, in control fibroblasts, the activation of p38MAPK phosphorylation showed constantly high levels until 24 h, which decreased after 4 d.

Taken together, this data are indicative of an important role for *Nd2* as an upstream inducer of cellular senescence and a SASP. The different activation of the p38MAPK pathway in fibroblasts of both mouse strains suggests that p38MAPK signaling is involved in this process. To further address this issue a transcriptome analysis was performed.

### Nd2 mediates its effects via p38MAPK signaling

To identify genes and pathways involved in mediating the anti-senescence effects of mutant *Nd2*, a transcriptome analysis of *Nd2*-mutant (*n* = 4) and control skin fibroblasts (*n* = 4) from 12-month-old mice was performed. This screen identified 766 genes that were significantly differentially expressed (upregulated or downregulated) between both mouse strains (Table S2). Further analysis of the downregulated genes in *Nd2*-mutant fibroblasts with DAVID Bioinformatics Resources 6.7 program [http://david.abcc.ncifcrf.gov/] revealed an enrichment of pathways mainly involved in cancer (fold enrichment 2.34) and MAPK signaling (fold enrichment 2.24) (Figure 4B). Next, we investigated whether the downregulated genes that are involved in “pathways in cancer” and the “MAPK signaling pathway” showed interactions with established mediators of senescence like p38MAPK, which showed in our immunoblot analysis specific differences in expression and activation (Figure 4A). For this purpose, the genes of the top pathways were analyzed with the bioinformatics tool STRING 9.1 (Search Tool for the Retrieval of Interacting Genes and Proteins; [20]). The main members of p38MAPK pathway (MAPK14 (p38α) MAPK11 (p38β) and MAPK12 (p38γ), although not differentially expressed, was included in this analysis (Figure 4C). Interestingly, the analysis revealed that 50% of the top enriched genes (involved in “MAPK signaling pathways” and “Pathways in cancer”) interact with p38MAPK. This suggested that the *Nd2*-induced pro-senescence effects in mouse skin fibroblasts are at least in part mediated by p38MAPK signaling. As mentioned, both *Nd2*-mutant and control cells showed comparable levels of p38α mRNA (Figure S2).

In the transcriptome analysis, we found that one half of the downregulated genes of enriched pathways (“MAPK signaling pathways” and “Pathways in cancer”) in *Nd2*-mutant fibroblast interact with the p38MAPK signaling pathway. The p38MAPK pathway is described as a crucial SASP regulatory pathway in the process of cellular senescence, which is activated by phosphorylation in response to acute cellular stress [16]. To test whether the reduced senescence in *Nd2*-mutant mice might involve p38MAPK, we inhibited p38MAPK with the chemical inhibitor SB203580 (SB). This potent inhibitor acts via prevention of ATP binding to the p38MAPK ATP binding pocket but leaves p38 phosphorylation unaffected [16].

**Figure 5:**
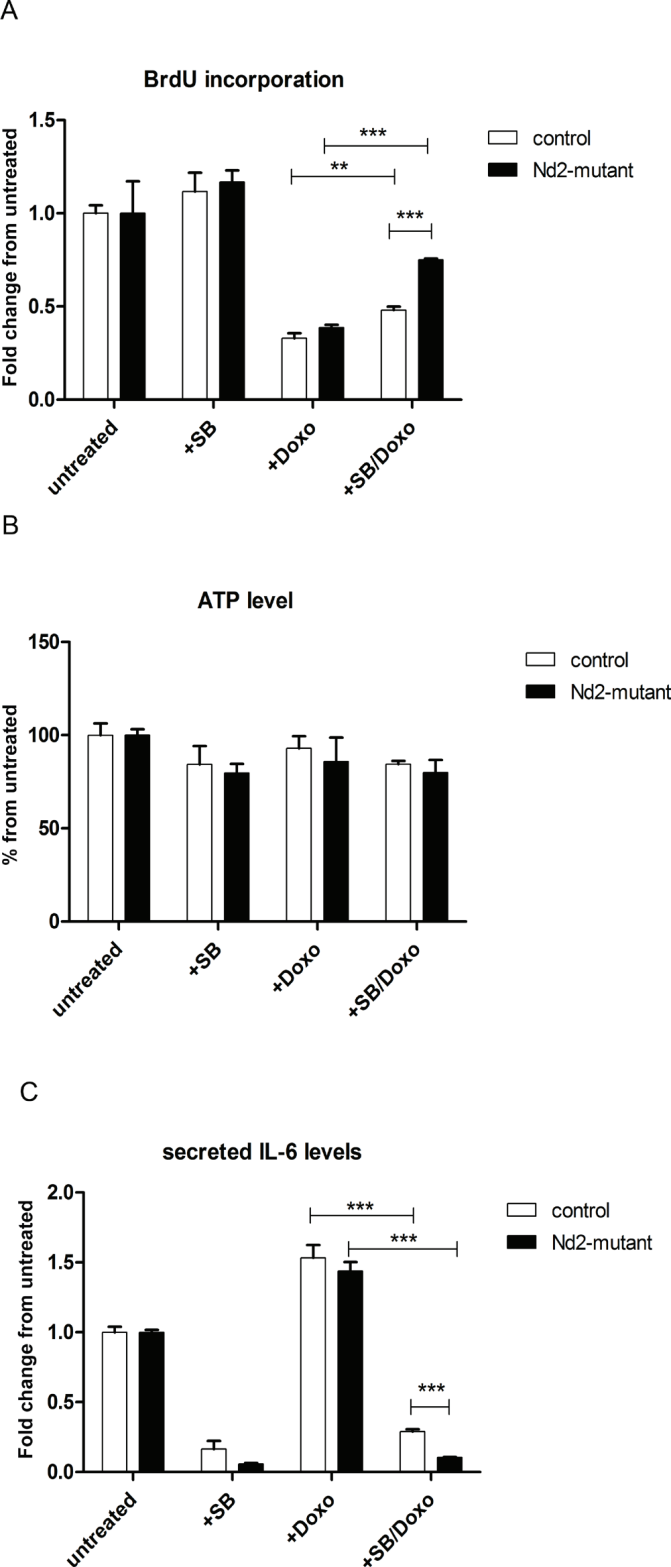
Influence of p38MAPK inhibition on expression of senescence markers in *Nd2*-mutant mouse fibroblasts **A.** Primary fibroblasts of 3-month-old mice were treated with doxorubicin and SB203580 alone or in combination. Cellular proliferation was measured by BrdU incorporation assay on day 4. **B.** Primary fibroblasts from skin of 3-month-old mice were treated with doxorubicin and SB203580 alone or in combination. ATP levels were measured on day 4. **C.** After treatment of fibroblasts from skin of 3-month-old mice with doxorubicin and SB203580 alone or in combination, cell supernatants were collected and analysed by ELISA for IL-6. The concentration of doxorubicin was 250 nM, exposure time was 1 h; SB concentration was 10 μM for continuous treatment. *n* = 3. Data are given as mean + SEM. *p < 0.05; **p < 0.01; ***p < 0.001.

For this purpose, fibroblasts from the *Nd2*-mutant and control strain were treated with SB203580. First, to identify the optimal treatment procedure, SA-β-gal assays were performed with skin fibroblasts from C57BL/6J mice treated with different concentrations of SB203580 (Figure S3). A concentration of 10 μM showed an effective reduction of the amount of SA-β-gal-positive cells by 50% after 4 days of continuous treatment. Thus, this concentration was used for further experiments. Next, fibroblasts were treated with doxorubicin to induce senescence and cellular proliferation was measured after 4 d. Doxorubicin treatment led to reduction of proliferation by 60% in fibroblasts of both strains. In both strains the proliferation measured with BrdU incorporation increased significantly by adding SB203580 to the doxorubicin treatment. Interestingly, the increase in proliferation in *Nd2*-mutant mouse fibroblasts is significantly higher as compared with control fibroblasts (Figure 5A).

To investigate the role of p38MAPK inhibition on mitochondrial function, ATP levels in fibroblasts of both mouse strains were analyzed (Figure 5B). We did not observe changes on ATP production after treatment with doxorubicin and SB203580 in skin fibroblasts of mutant and control fibroblasts compared with untreated control. This suggests that inhibition of p38MAPK has no influence of the production of ATP by the respiratory chain.

IL-6 is a major cytokine of the SASP. Cytokine secretion of fibroblasts was measured by ELISA (Figure 5C). To determine whether *Nd2* has an impact on the induction of the SASP, we treated skin fibroblasts from *Nd2*-mutant and control mice with doxorubicin and SB203580. Under doxorubicin treatment alone, secretion of IL-6 increased in both strains as compared with untreated control. Interestingly, the additional inhibition of p38MAPK by SB203580 treatment led to a reduction of IL-6, which was even significantly stronger in *Nd2*-mutant fibroblasts as compared with control fibroblasts.

Taken together, these results provide strong evidence that p38MAPK is a downstream effector of *Nd2*-induced cellular senescence. The inhibition of p38MAPK in *Nd2*-mutant cells, but not in control cells rescues the cells from senescence features such as reduced proliferation, reduced viability and enhanced IL-6 secretion induced by genotoxic stress-inducing agent doxorubicin.

### Epidermal thickness of *Nd2*-mutant mice

**Figure 6:**
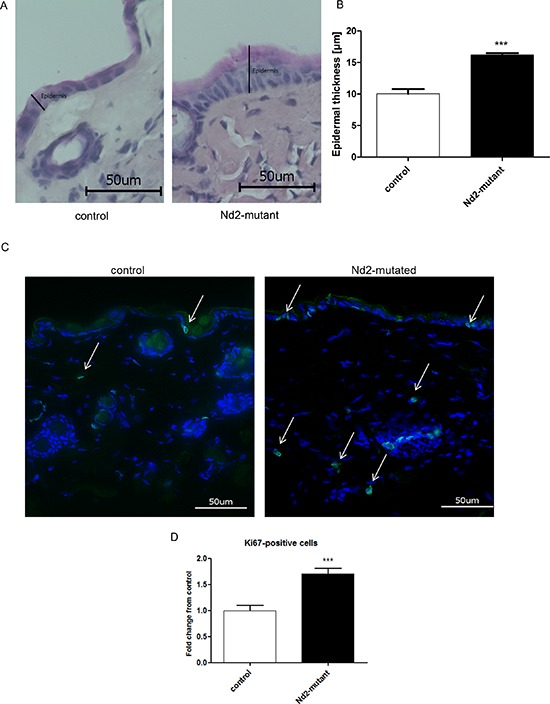
Measurement of epidermal thickness and Ki67 staining of the skin of *Nd2*-mutant mice **A.** Representative photomicrographs of H&E staining of skin sections from control (*n* = 4) and *Nd2*-mutant (*n* = 4) mice, aged 12 months. **B.** Quantification of the thickness of the epidermis in skin sections from control (*n* = 4) and *Nd2*-mutant (*n* = 4) mice using BZ II analyzer software. ***p < 0.001. Data expressed as mean ± SEM. **C.** Representative photomicrographs of Ki67 immunofluorescence staining (green) of skin sections from control (*n* = 5) and *Nd2*-mutant mice (*n* = 5), aged 12 month. DAPI was used for counterstaining of cell nuclei. **D.** Quantification of Ki67-positive cells in skin sections from control and Nd2-mutant mice using Image J software. ***p < 0.001. Data are expressed as mean ± SEM.

Next, we wondered whether the reduced cellular senescence effects of *Nd2*-mutant fibroblasts translate into reduced aging of the skin *in vivo*. The common *in vitro* marker SA-β-galactosidase showed contradictory results [21, 22] *in vivo*. For this reason, other senescence markers were preferred like epidermal thickness and the number of Ki67-positive cells in skin of mutated and control mice. Indeed, 12-month-old *Nd2*-mutant mice showed a thicker epidermis relative to control mice, whose skin was more atrophic (Figure 6A and 6B). This phenomenon is due to a higher number of epidermal keratinocyte layers consistent with the higher proliferation rate of epidermal keratinoyctes in these mice.

Finally, the skin of 12-month-old *Nd2*-mutant and control mice was stained for the proliferation marker Ki67 (Figure 6C and 6D). The nuclei were counterstained with DAPI. The Ki67-positive cells were determined by ratio of Ki67-positive/Ki67-negative cells in the epidermis and dermis excluding hair follicles. In *Nd2*-mutant mouse skin, the amount of Ki67-positive cells was significantly higher than in control skin. The higher amount of Ki67-positive cells and the thicker epidermis in *Nd2*-mutant mice is strongly supported the *in vitro* data.

## DISCUSSION

Mitochondrial dysfunction, and in particular impairment of the mitochondrial oxidative phosphorylation (OXPHOS) system, has been shown to result in various degenerative diseases and cancers [23, 24]. Deficits of the respiratory chain complexes (particularly complexes I, II, IV or V) are responsible for the neurodegenerative disorder Leigh syndrome or Leigh-like syndrome. Patients with Leigh syndrome are carrying specific point mutations in the mitochondrially encoded subunits 1–6 of NADH dehydrogenase, cytochrome c oxidase and ATPase [25]. Mutations in complex I to V were also found in different human cancers such as prostate, pancreas, colon, and breast cancer [23]. However, genetic alterations in these genes could also confer resistance to other diseases. For example, a mutation in the mitochondrial gene NADH dehydrogenase subunit 2, a cytosine to adenine transversion at position nt5178 (C5178A) resulting in a leucine to methionine substitution has been associated with the reduction of the incidence of diseases such as atherosclerosis, myocardial infarction and type 1 diabetes in Japanese patients [14].

By using conplastic mouse strains, we established new mouse models to analyze the influence of specific mitochondrial gene variations in mitochondrial respiratory chain complexes on cellular senescence and skin aging. We used isolated skin fibroblasts from mouse strain C57BL/6J-mt^ALR/LtJ^ (*Nd2*-mutant) and from C57BL/6J-mt^AKR/J^ as control in all experiments. The two strains only differ in one mutation. The mutant strain carries the mutation in NADH dehydrogenase subunit 2 at position nt4738. This mutation leads to an adenine to cytosine exchange. We first analyzed several parameters of basal mitochondrial function and identified a difference in complex I activity, ATP- and ROS production between the strains. Fibroblasts from the *Nd2*-mutant strain showed a significantly higher mitochondrial complex I activity. This higher activity was associated with significantly enhanced ATP production and reduced ROS production with similar Nd2 protein expression levels. According to the gradual ROS response hypothesis [26], while ROS at lower levels is considered beneficial as a mediator of stress response and repair, ROS at higher levels are involved in organismal aging as oxidative damage in cells has been shown to limit the life span of organisms [27]. ROS have also been shown to enhance cellular senescence and organismal aging [28]. In line with our findings, several studies in different organisms have shown a negative relationship between mitochondrial ROS production and lifespan. For example, Lambert et al. isolated heart mitochondria from mouse, rat, white-footed mouse, naked mole-rat, and a series of other animal species and showed that maximum life span was negatively correlated with hydrogen peroxide production during reverse electron transport in mitochondrial respiratory chain complex I [28]. Although, complex I and complex III are considered to be two major sources of ROS production in mitochondria, there is evidence that complex I accounts for majority of the ROS generated in intact mammalian mitochondria [29, 30]. Moreover, it has been postulated that decreased activity of complex I in brain mitochondria from Parkinson's patient results directly from oxidative damage of its subunits [31]. In another study, comparing the type 1 diabetes-prone non-obese diabetic (NOD) mouse strain with the alloxan-resistant (ALR) mouse strain, the NADH dehydrogenase 2 gene was found to be involved in conferring resistance to diabetes and exert an influence on ROS generation. Mitochondria from ALRmt^NOD^ mice carry the cytosine-containing allele mt-Nd2^c^, which resulted in increased ROS production. Our findings agree with a damaging role of ROS, suggesting that its level is sufficiently high to cause (oxidative) damage in our experimental system. Accordingly, in our case the mitochondria with the adenine containing allele (mt-Nd^a^) that results in a leucine to methionine transition yield lower ROS levels, which is beneficial, in line with [4, 14].

To determine whether higher complex I activity and reduced ROS levels in the *Nd2*-mutant strain have any influence on cellular survival and senescence; we analyzed fibroblast proliferation and expression of senescence markers like SA-β-gal. Indeed, *Nd2*-mutant skin fibroblasts showed significantly increased ability to resist cellular senescence characterized by reduced SA-β-gal activity, showed enhanced proliferation and reduced secretion of senescence-associated cytokine. We hypothesize that the reduced senescence phenomena in *Nd2*-mutant cells are a result of lower ROS production as a consequence of high complex I activity.

p38MAPK is described as an important promoter of cellular senescence mediated by NF-κB signaling [16]. However, the interplay between p38MAPK and complexes of the OXPHOS system is largely unknown. Interestingly, our transcriptome analysis comparing *Nd2*-mutant and control mice showed a reduced expression of different genes in *Nd2*-mutant mice, a majority of which were found to interact with p38MAPK. On the protein level, the activation/expression of p38MAPK after doxorubicin treatment was found to be transient in *Nd2*-mutant mouse at 2 h but stable until 24 h in control fibroblasts. This suggested that *Nd2*-induced cellular senescence is at least in part mediated through p38MAPK.

Recent studies have demonstrated that ROS can induce or mediate the activation of p38MAPK [32]. We hypothesized that low intracellular ROS levels in *Nd2*-mutant mouse fibroblasts lead to a low activation of the p38MAPK pathway with the consequence of reduced IL-6. To further address this, we used the p38MAPK inhibitor SB203580 to investigate the impact of an activated p38MAPK pathway on IL-6. Pharmacological inhibition of p38MAPK activation with SB203580 could prevent cells from entering the process of senescence [33, 16]. p38MAPK inhibition had stronger effects on senescence-associated features such as reduced proliferation, and IL-6 secretion induced by the DNA damaging agent doxorubicin in *Nd2*-mutant mouse fibroblasts, than in control fibroblasts. These results supported our findings that the *Nd2*-mutation in fibroblasts leads to an attenuated activation the p38MAPK pathway, finally resulting in reduced senescence. In line with our findings, p38MAPK inhibition by SB203580 was found to mitigate components of SASP in an earlier study [16]. In this study, radiation-induced senescent HCA2 fibroblasts treated with SB203580 showed reduced secretion of IL-6, IL-8 and GM-CSF. The study also showed that p38MAPK inhibition repressed the pro-invasive effects of senescent cells [16].

In an earlier study, mtDNA from 37 Japanese centenarians and 43 controls was analysed [12]. Mutations at position nt5178 in the *ND2* gene were more frequently observed in centenarians than in controls [12]. These findings suggested that this mutation in *ND2* is related to longevity.

The accumulation of cellular and oxidative damage by ROS is not the only mechanism which may explain mechanism underlying senescence and limitation of life span. There is strong evidence that the nutrient-sensing molecular mTOR (target of rapamycin) pathway influences ROS generation and may play a central role in aging and nutritional control of life span [34]. Recent studies showed that the inhibition of mTOR with rapamycin extends life span in mice and prevents age-related diseases [35]. In flies and yeast, inhibition of mTOR may lead to increased antioxidant defenses [36]. There are different links between ROS and mTOR pathway. On the one hand, ROS can activate mTOR and on the other hand TOR can increase or decrease the ROS production [34]. In studies with human cells hydrogen peroxide activated the PI3-K (phosphatidylinositol-3-kinase)/mTOR/S6K (ribosomal protein S6 kinase beta-1) pathway [37, 38]. It was further shown that ROS production was decreased in primary rat hepatocytes after mTOR inhibition with rapamycin [39]. Taken together, p38MAPK and PI3/AKT/mTOR pathway appear to be strongly involved in cellular senescence.

In conclusion, our study comprehensively demonstrates that a mutation in the *ND2* gene leads to reduced activation of p38MAPK pathway, likely mediated by lower ROS levels. This interplay of the *ND2* mutation, reduced ROS production and p38MAPK activation could be protective against senescence and other pathological conditions, which finally result in a prolonged lifespan of skin fibroblasts and, maybe, even the whole organism.

## METHODS

### Mice

Conplastic mice were generated by crossing females from the mitochondrial donor strain to males with a C57BL/6J background [15]. In each generation, the female offspring were backcrossed to males. After 10 backcrosses, the offsprings were regarded as conplastic strains with the nuclear genome of the recipient strain and the mitochondrial genome from the donor strain. Animals were housed at the animal facility at the University of Lübeck. For this study, we used C57BL/6J-UCP2−/−, C57BL/6J-mt^FVB/NJ^, C57BL/6J-mt^129S1/SvlmJ^ C57BL7/6J-mt^ALR/LtJ^ as *Nd2*-mutant strain and C57BL/6J-mt^AKR/J^ as controls.

### Cell culture

Primary skin fibroblasts were isolated from mutant and control mice using a standard dissociation protocol adapted to the Liberase DL Research Grade (low dispase) protocol of Roche Diagnostics (Mannheim, Germany). Skin biopsies were cut into small pieces and added to Dulbecco's Modified Eagle's Medium (DMEM) containing Liberase DL Research grade with a final concentration of 1.3 U/mL and were incubated for 2 hours. Isolated fibroblasts were cultured for 5 days in DMEM, supplemented with 10% FCS (Biochrom GmbH, Berlin, Germany), Penicilin/Streptavidin, Mycokill (PAA Laboratories, Pasching, Austria) in 5% CO_2_ at 37°C. Before experiments, cells were cultured for 2 days in Mycokill-free medium. For all experiments, cells from passage 1 were used.

### Senescence-associated β-galactosidase assay

Cells were fixed and stained for SA-β-gal using Senescence β-galactosidase Staining Kit from Cell Signaling (Frankfurt/Main, Germany). The ratio of the number of positive blue cells and the total number of cells was used to determine the percentage of SA-β-gal-positive (senescent) cells.

### Determination of ROS

Intracellular ROS levels were determined with 2′, 7′-dichlorodihydrofluorescein diacetate (DCFH-DA; Sigma-Aldrich, Hamburg, Germany). Fibroblasts were incubated for 30 min with 50 μM DCFH-DA after which the cells were washed with PBS. Oxidized DCF was measured at excitation wavelength of 488 nm and emission wavelength at 525 nm. Data were normalized to internal control (8- to 12-weeks-old male C57BL/6J mouse fibroblasts).

### Determination of ATP

ATP determination was performed using the CellTiter-Glo^®^ Luminescent Cell Viability Assay following the manufacturer's instructions. Data were calculated based on the standard values generated from known concentrations of ATP dissolved in dH_2_O (Promega, Mannheim, Germany).

### Cell proliferation assay

For measurement of cell proliferation, a 5-bromo-2′-deoxy-uridine cell proliferation ELISA was used according to the manufacturer's instruction (Roche Diagnostics, Mannheim, Germany). Cells were labeled with 10 μM BrdU labeling reagent for 2 hours. Data were normalized to internal control (C57BL/6J primary skin fibroblasts).

### ELISA

IL-6 (ebioscience GmbH, Frankfurt/Main, Germany) and KC (mouse homologue of IL-8) (PromoCell, Heidelberg, Germany) concentrations were measured by ELISA following manufacturer's instructions. Supernatants were assayed as triplicates and values were calculated to standard values made from known concentration of IL-6 or KC (IL-8). Untreated values were subtracted from doxorubicin-treated values to calculate the secretion of the indicated cytokine.

### Western blot

Immunoblots were performed as described in [40]. The following primary antibodies were used: anti γ-H2A.X (phospho-histone H2A.X, sc-101696, Santa Cruz Biotechnology, Heidelberg, Germany), anti-p38MAPK (#9212), anti-phospho-p38 (3D7) (#9215), anti-phospho-p53 (#9284), all obtained from Cell Signaling Technology. For loading control, anti-ribosomal protein L26 (Sigma-Aldrich, Hamburg, Germany) was used. The following secondary antibodies were used: IRDye 680LT goat anti rabbit IgG (926–68021) and IRDye 800CW goat anti-mouse IgG (926–32210) (LI-COR Biosciences, Bad Homburg, Germany). Signals were measured with LI-COR Odyssey^®^ scanner and quantified with LI-COR Odyssey^®^ software 3.0 (LI-COR Biosciences).

### Histopathology

Skin samples were cut into sections of 8 μM thicknesses and stored at −80°C. Sections were fixed in ice-cold acetone and stained with hematoxylin and eosin (H&E). Epidermal thickness was measured by using BZ II analyzer software (Keyence, Osaka, Japan).

### Immunofluorescence staining

Skin biopsies were cut into 8 μM sections and stored at −80°C. Frozen sections were fixed and incubated with primary antibodies 2 h in PBS/0.1% Tween 20/0.3% BSA. Primary antibody from Bethyl Laboratories, Inc. (Ki-67, 1:400; Montgomery, Texas, USA) was used. Cell nuclei were stained with 4′, 6-diamidino-2-phenylindole (DAPI). Images were acquired with a BZ-9000E microscope camera and quantitated using ImageJ with the graylevel watershed algorithm (http://bigwww.epfl.ch/sage/soft/watershed/).

### Isolation of mitochondria

Mitochondria were isolated from primary skin fibro blasts using the Mitochondria Isolation Kit for tissue and cultured cells. According to the manufacturer's instructions (BioVision Inc, Milpitas, California, USA) 2 × 10^7^ cells were used for isolation and protein concentrations were determined by bicinchoninic acid protein assay (BCA, Thermo Fisher Scientific, Bonn, Germany).

### Complex I activity assay

Complex I activity was determined by measuring the oxidation of NADH to NAD^+^ at 340 nm. The assay medium contained K_2_HPO_4_ (25 mM; pH 7.4), 5 mM MgCl_2_, 0.25% BSA, 3.7 μM antimycin and 2 mM KCN. A base line was established after addition of 5.7 mM NADH and isolated mitochondria of skin fibroblasts (31 μg). The reaction was started by the addition of 2.8 mM CoQ_1_. The rate of oxidation of NADH was recorded for 5 min. Rotenone (0.36 mM), an inhibitor of complex I was added to determine the rotenone insensitive activity. Complex I activity was calculated by subtraction of the reaction rates in the presence and absence of rotenone.

### Quantitative real time PCR

Total RNA from cells was extracted using the RNeasy MiniKit (Qiagen, Hilden, Germany). cDNA was transcribed with the High capacity cDNA-RT-Kit (Life Technologies, Carlsbad, CA, USA) according to manufacturer's instructions. For determination of MAPK14 gene expression Real-time TaqMan™ PCR was performed with GoTaq^®^ qPCR Master Mix according to manufacturer's instructions. For PCR amplification and signal detection the StepOne™ Real-Time PCR system according to manufacturer's instructions. Ct values were normalized to RPL26 and differences of gene expression were calculated with the DDCt method.

### Microarray analysis

Whole genome cDNA microarray analyses were performed using skin fibroblasts isolated from C57BL/6J-mt^ALR/LtJ^ (*Nd2*-mutant) and C57BL/6J-mt^AKR/J^ (control) mouse strains (four samples per group). RNA integrity and concentration was examined on an Agilent 2100 Bioanalyzer (Agilent Technologies, Palo Alto, CA, USA) using the RNA 6.000 LabChip Kit (Agilent Technologies) according to manufacturer's instructions. Illumina BeadChip analysis was conducted at the microarray core facility of the Interdisziplinäres Zentrum für klinische Forschung (IZKF) Leipzig (Faculty of Medicine, University of Leipzig).

250 ng RNA per sample were ethanol precipitated with GlycoBlue (Life technologies, Carlsbad, CA, USA) as carrier and dissolved at a concentration of 100–150 ng/μl prior to probe synthesis using the TargetAmp™- Nano Labeling Kit for Illumina Expression BeadChip (Epicentre Biotechnologies, Madison, WI, USA). 750 ng of cRNA were hybridized to MouseRef-8 v2.0 Expression BeadChips (Illumina, San Diego, CA, USA) and scanned on the Illumina HiScan instrument according to manufacturer's specifications. Raw data of 25, 600 probes was extracted and quantile normalized by Illumina GenomeStudio^®^. Expression values were background subtracted if necessary. We considered probes as present for a detection *p*-value < 0.01. Thereafter, we used Student's *t*-tests to identify differentially expressed genes. The results were corrected for multiple testing using the Benjamini-Hochberg method. Genes were considered significantly differently expressed if *p* < 0.05.

The data was processed and analysed using the Bioconductor packages lumi and limma [41, 42] in the statistical programming environment R. Finally, differentially expressed genes where subjected to a functional enrichment analysis using the DAVID Bioinformatics Resources 6.7 program (http://david.abcc.ncifcrf.gov/). We used the default parameters to identify enrichment of candidate gene in KEGG pathways.

Geo accession number: http://www.ncbi.nlm.nih.gov/geo/query/acc.cgi?acc=GSE63551.

### Statistical analysis

Data are expressed as mean ± SEM. Standard analysis was performed with GraphPad Prism 5 (GraphPad, Inc, San Diego, CA) using Student's *t*-test. A *p*-value of < 0.05 was considered to be statistically significant.

## SUPPLEMENTAL FIGURES AND TABLES


