# Supplementary material for: A mutation in the NADH-dehydrogenase subunit 2 suppresses fibroblast aging
Source: Oncotarget. 2015 Mar 24;6(11):8552–66. doi: 10.18632/oncotarget.3298 (PMC4496166; doi:10.18632/oncotarget.3298)
Supplement: Supplementary file 1 [file oncotarget-06-8552-s001.pdf]

## SUPPLEMENTAL FIGURES AND TABLES

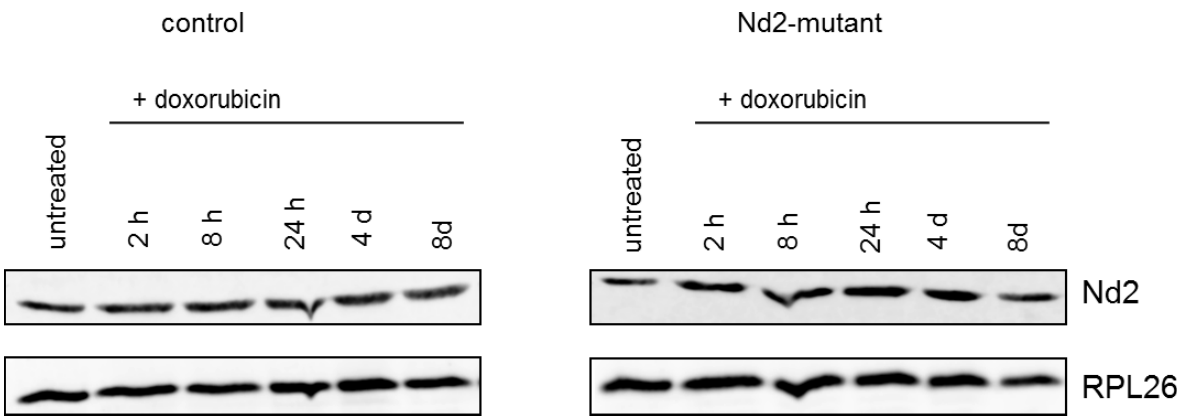


**Supplementary Figure S1: Nd2 protein expression of after doxorubicin treatment.** Fibroblasts of *Nd2*-mutant and control mice were treated with 250 nM doxorubicin and whole cell lysates collected at the indicated days thereafter. Lysates were pooled from three different mouse fibroblasts. RPL26 was used as loading control.

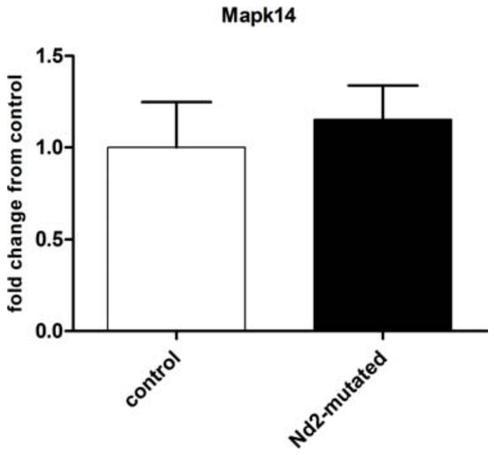


**Supplementary Figure S2: p38α mRNA expression of Nd2 mutant and control fibroblasts.** RNA was extracted from fibroblasts of Nd2-mutant and control mice. For Mapk14 (p38α) gene expression Real-time TaqMan™ PCR was performed. RPL26 was used for normalization. RNA from three different mice were pooled and data are given as mean ± SEM.

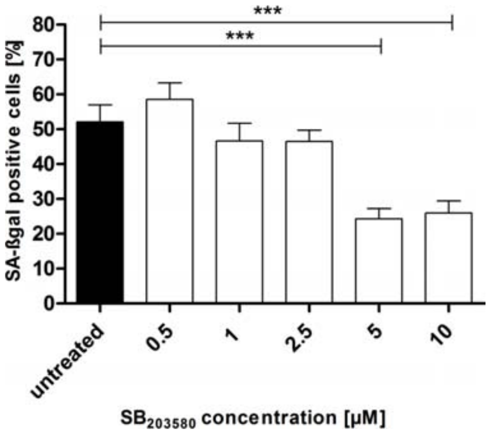


**Supplementary Figure S3: SA-β-galactosidase activity after treatment of fibroblasts with p38MAPK inhibitor SB203580.** Primary fibroblasts from C57BL/6J mice were continuously treated with different concentration of SB203580 for 4 days. The cells were fixed and stained for SA-β-gal. At each concentration eight fields were randomly counted. The number of positive blue cells was divided by the total number of counted cells resulting in the percentage of galactosidase-positive cells. $n = 3$, ***$p < 0.001$. Data are expressed as mean ± SEM.

**Supplementary Table S1. List of mtDNA mutations in conplastic inbred strains and uncoupling protein 2-knockout strain**

| Position (bp) | AKR/J | ALR/LtJ | FVB/NJ | 129S1/ SvlmJ | UCP2-/- | Gene | Amino acid | Complex |
|---|---|---|---|---|---|---|---|---|
| **4738** | C | A | | | | mt-*Nd2* | Leu-Met | I |
| **7778** | G | | T | | | mt-*ATP8* | Asp-Tyr | V |
| **15124** | A | | | G | | mt-*Cytb* | Ile-Val | III |
| **UCP2** | + | | | | − | *UCP2* | ko | |

+UCP2 gene
−UCP2 knockout; bp basepair

**Supplementary Table S2. Downregulated genes of *Nd2*-mutant mouse fibroblasts versus control mouse fibroblasts determined with MouseRef-8 v2.0 Expression BeadChips**

| Coefficient ALR vs AKR | Gene symbol | *p*-value ALR vs AKR |
|---|---|---|
| −1708,064 | Actg2 | 0,04567 |
| −1632,127 | Igfbp7 | 0,04658 |
| −1354,683 | NR_003619 | 0,01443 |
| −1301,584 | Rpl3 | 0,00972 |
| −1286,193 | Actb | 0,03539 |
| −1131,224 | Rps24 | 0,02063 |
| −918,299 | Lmna | 0,04474 |
| −637,971 | NR_003363 | 0,02683 |
| −618,307 | Timp3 | 0,00979 |
| −599,207 | Igfbp5 | 0,01402 |
| −595,307 | Psmd4 | 0,04849 |
| −573,103 | Hnrnpm | 0,01071 |
| −544,571 | Eif4a2 | 0,04196 |
| −535,892 | Cd44 | 0,01201 |
| −458,857 | Aprt | 0,01229 |
| −450,589 | Prdx1 | 0,04024 |
| −431,895 | Hdlbp | 0,00011 |
| −373,136 | Plekhg3 | 0,02941 |
| −352,525 | Rangap1 | 0,00623 |
| −339,443 | Npm3 | 0,03677 |
| −336,746 | Atf5 | 0,00867 |
| −323,739 | Degs1 | 0,00991 |
| −323,191 | Glt25d1 | 0,03855 |
| −315,089 | Ywhab | 0,00118 |
| −305,37 | Cdipt | 0,03127 |
| −286,92 | Tmem109 | 0,0215 |
| −283,196 | Mdm2 | 0,02058 |
| −273,647 | Pon2 | 0,02062 |
| −266,334 | Tgm2 | 0,00635 |
| −255,651 | Nap1l4 | 0,00117 |
| −255,462 | Lmo4 | 0,01974 |
| −232,792 | Frmd6 | 0,04835 |
| −231,559 | Cope | 0,04006 |
| −228,671 | Tmem111 | 0,03223 |
| −220,399 | Srf | 0,02079 |

(*Continued*)

| Coefficient ALR vs AKR | Gene symbol | *p*-value ALR vs AKR |
|---|---|---|
| −212,317 | Ppid | 0,00713 |
| −206,313 | Ttc13 | 0,01597 |
| −191,04 | Hbs1l | 0,03213 |
| −186,879 | Tmem132a | 0,03591 |
| −186,57 | RP23-195K8 | 0,04452 |
| −181,345 | AW555464 | 0,01263 |
| −180,191 | Map2k2 | 0,04844 |
| −178,48 | Mrpl20 | 0,03988 |
| −172,609 | Lias | 0,01028 |
| −171,636 | 6430706D22 | 0,02258 |
| −166,359 | Dhrs7b | 0,00097 |
| −165,234 | Endod1 | 0,01132 |
| −163,767 | Aip | 0,00005 |
| −161,191 | Cggbp1 | 0,02841 |
| −161,125 | Tgfb3 | 0,02565 |
| −158,407 | Slc35b2 | 0,04079 |
| −153,532 | Elavl1 | 0,03206 |
| −153,356 | Itpr1 | 0,02112 |
| −151,817 | Phf5a | 0,02703 |
| −148,026 | Klhdc4 | 0,04739 |
| −147,624 | Dkk3 | 0,03368 |
| −147,384 | Mark3 | 0,03908 |
| −141,919 | Dbndd2 | 0,03641 |
| −140,249 | Htra1 | 0,00883 |
| −137,462 | Picalm | 0,02672 |
| −134,981 | Dlst | 0,01553 |
| −134,251 | G3bp1 | 0,01797 |
| −133,567 | Mmp17 | 0,00682 |
| −132,608 | Efnb1 | 0,04252 |
| −131,732 | Preb | 0,02896 |
| −127,676 | Smt3ip2 | 0,01785 |
| −125,311 | Emcn | 0,00931 |
| −123,524 | Itprip | 0,02166 |
| −122,758 | Pls3 | 0,04253 |
| −122,205 | Prmt5 | 0,00388 |
| −119,881 | Creld1 | 0,04699 |
| −118,117 | Bclaf1 | 0,0356 |

(*Continued*)

| Coefficient ALR vs AKR | Gene symbol | *p*-value ALR vs AKR |
|---|---|---|
| −117,83 | Vars | 0,01157 |
| −113,761 | Mrpl2 | 0,00657 |
| −113,33 | Sdc3 | 0,00109 |
| −111,554 | Kif1b | 0,00522 |
| −110,76 | Rgs4 | 0,01513 |
| −110,561 | Fbxw5 | 0,00874 |
| −109,066 | Mars | 0,00066 |
| −105,128 | Rpp21 | 0,00924 |
| −105,083 | Cox10 | 0,01845 |
| −104,725 | Fam160a2 | 0,00401 |
| −101,66 | Tmbim4 | 0,00287 |
| −100,154 | Vps37b | 0,02247 |
| −98,342 | Ppp1r10 | 0,01588 |
| −98,269 | Zfp131 | 0,04936 |
| −97,094 | Dpagt1 | 0,03593 |
| −96,84 | Prkd2 | 0,04849 |
| −96,406 | Ppm1b | 0,04791 |
| −96,326 | Ate1 | 0,00648 |
| −93,034 | Cd59a | 0,00975 |
| −91,477 | Cep120 | 0,02257 |
| −91,245 | Pycr2 | 0,04123 |
| −90,289 | Has1 | 0,03013 |
| −89,765 | Btbd3 | 0,01383 |
| −89,632 | Dpysl3 | 0,01311 |
| −89,035 | Smc1a | 0,03881 |
| −88,815 | B020018G12 | 0,02903 |
| −87,468 | Mcam | 0,03495 |
| −85,587 | Mfn1 | 0,00584 |
| −83,634 | Ednrb | 0,04054 |
| −83,312 | Rrp12 | 0,0353 |
| −81,763 | Lims2 | 0,03008 |
| −80,306 | Plvap | 0,04397 |
| −80,252 | Supt5h | 0,041 |
| −79,827 | Inpp5a | 0,00461 |
| −79,047 | Zdhhc12 | 0,02736 |
| −78,664 | 1500010J02 | 0,02552 |
| −78 | Flrt3 | 0,01783 |

(*Continued*)

| Coefficient ALR vs AKR | Gene symbol | *p*-value ALR vs AKR |
|---|---|---|
| −77,829 | Mogs | 0,02957 |
| −74,422 | Npepl1 | 0,02633 |
| −70,438 | B230339M05 | 0,00058 |
| −70,181 | Lims2 | 0,01213 |
| −69,84 | 0610007P22 | 0,0402 |
| −69,795 | Wdr75 | 0,04091 |
| −67,986 | Rbm9 | 0,03614 |
| −66,727 | AA536749 | 0,01998 |
| −66,138 | Ncoa5 | 0,03085 |
| −65,955 | Sltm | 0,03565 |
| −65,668 | Gart | 0,01606 |
| −65,249 | Dus1l | 0,02545 |
| −65,131 | Mcam | 0,04444 |
| −64,132 | Gpc4 | 0,0478 |
| −63,685 | Mcm10 | 0,02521 |
| −63,488 | Gpn1 | 0,02862 |
| −62,86 | Dazap1 | 0,02732 |
| −62,494 | Ush2a | 0,01904 |
| −62,319 | Shmt2 | 0,01878 |
| −62,29 | Stx12 | 0,05 |
| −60,023 | Smek2 | 0,04752 |
| −59,898 | Acer3 | 0,01473 |
| −59,178 | Tmem68 | 0,01043 |
| −58,521 | Pop5 | 0,04424 |
| −58,355 | 2410001C21 | 0,04233 |
| −58,194 | Bmp4 | 0,01613 |
| −58,136 | Plaur | 0,01365 |
| −57,725 | Helz | 0,00549 |
| −57,678 | Styx | 0,01719 |
| −57,626 | Tuft1 | 0,04208 |
| −57,564 | Hr | 0,00413 |
| −55,934 | Il6 | 0,015 |
| −55,89 | Creb3l2 | 0,02089 |
| −55,752 | 2310003H01 | 0,03125 |
| −55,008 | Cdsn | 0,01768 |
| −54,28 | Prkag1 | 0,024 |
| −53,822 | Itga3 | 0,02841 |

(*Continued*)

| Coefficient ALR vs AKR | Gene symbol | *p*-value ALR vs AKR |
|---|---|---|
| −53,65 | Smek2 | 0,0333 |
| −53,641 | Noc4l | 0,0174 |
| −53,588 | Cnot10 | 0,01452 |
| −52,4 | Lama1 | 0,03408 |
| −52,039 | Metrn | 0,03027 |
| −52,017 | Mybl2 | 0,01202 |
| −51,566 | Psmg2 | 0,01483 |
| −50,685 | Derl2 | 0,00776 |
| −50,648 | BC011248 | 0,022 |
| −49,742 | Dusp11 | 0,03987 |
| −49,687 | Ascc1 | 0,03239 |
| −49,179 | Yeats4 | 0,00544 |
| −49,07 | Ift81 | 0,04535 |
| −48,5 | Ccdc134 | 0,02345 |
| −48,49 | Rnf8 | 0,01363 |
| −48,419 | Timm50 | 0,02229 |
| −48,204 | Snupn | 0,01245 |
| −47,884 | Fip1l1 | 0,02926 |
| −47,498 | Mipep | 0,0386 |
| −47,398 | 5430437P03 | 0,01932 |
| −46,552 | Tbc1d10a | 0,0287 |
| −45,912 | Ecsit | 0,02965 |
| −45,872 | Slc25a25 | 0,00644 |
| −45,781 | Kif15 | 0,02352 |
| −45,247 | Rce1 | 0,0445 |
| −45,246 | Rell1 | 0,01686 |
| −45,078 | Sh3rf1 | 0,00658 |
| −44,478 | Pde4dip | 0,00961 |
| −44,297 | Mrvi1 | 0,00126 |
| −43,992 | Osbpl3 | 0,02715 |
| −42,684 | Slco3a1 | 0,04722 |
| −41,808 | 1300001I01 | 0,01884 |
| −41,718 | Pafl | 0,00458 |
| −41,103 | Tnn | 0,01826 |
| −40,628 | D12Ertd553 | 0,02226 |
| −40,514 | Cep164 | 0,03465 |
| −40,223 | Limk2 | 0,04451 |

(*Continued*)

| Coefficient ALR vs AKR | Gene symbol | *p*-value ALR vs AKR |
|---|---|---|
| −40,028 | Smad5 | 0,00262 |
| −39,651 | AI847670 | 0,00412 |
| −39,05 | Ahi1 | 0,02232 |
| −38,671 | Dmtf1 | 0,02877 |
| −38,595 | Znhit2 | 0,02531 |
| −38,194 | Cc2d1a | 0,03814 |
| −38,139 | Acbd6 | 0,02939 |
| −37,889 | E130016E03 | 0,03475 |
| −37,274 | Prkrip1 | 0,03891 |
| −37,08 | D5Ertd579e | 0,00965 |
| −37,069 | 5133401N09 | 0,00541 |
| −36,952 | Slc30a6 | 0,0448 |
| −35,297 | Rtel1 | 0,0491 |
| −35,276 | B230339M05 | 0,02783 |
| −35,042 | Jag1 | 0,01705 |
| −33,786 | Mgll | 0,04736 |
| −33,549 | Camk2n1 | 0,04751 |
| −33,084 | Git2 | 0,01976 |
| −32,65 | Hoxa7 | 0,03644 |
| −32,57 | Rad54l | 0,04811 |
| −32,002 | Rmnd1 | 0,04851 |
| −31,476 | Krt7 | 0,04304 |
| −31,327 | Trps1 | 0,04061 |
| −31,13 | Fam134b | 0,03598 |
| −30,592 | Trpv2 | 0,02716 |
| −30,264 | Clip1 | 0,01058 |
| −29,99 | Myo1b | 0,01573 |
| −29,638 | Epn2 | 0,01752 |
| −29,602 | Rab11fip3 | 0,04968 |
| −29,484 | Cyp4a12a | 0,02269 |
| −29,195 | Trrap | 0,04104 |
| −29,184 | Orai1 | 0,03426 |
| −29,037 | Pold3 | 0,02189 |
| −29,011 | Tgfb2 | 0,03761 |
| −28,985 | R3hdm2 | 0,02513 |
| −28,82 | Moxd1 | 0,02679 |
| −28,574 | Pde1b | 0,02885 |

(*Continued*)

| Coefficient ALR vs AKR | Gene symbol | *p*-value ALR vs AKR |
|---|---|---|
| −27,947 | Mecr | 0,00938 |
| −27,662 | Atp10d | 0,02417 |
| −27,382 | Ermp1 | 0,04755 |
| −27,174 | Gdpd5 | 0,01479 |
| −27,12 | Cadm1 | 0,03182 |
| −26,947 | Fadd | 0,00559 |
| −26,778 | Tarbp2 | 0,00087 |
| −26,526 | Mgll | 0,02515 |
| −26,47 | Gpaa1 | 0,01756 |
| −25,424 | Klf5 | 0,03272 |
| −25,373 | Timm44 | 0,0492 |
| −25,223 | Acer3 | 0,02933 |
| −25,144 | Ethe1 | 0,04234 |
| −24,723 | Tinagl1 | 0,00822 |
| −24,509 | Zfand2b | 0,01463 |
| −24,405 | Cacna1g | 0,04469 |
| −24,152 | Dlgap5 | 0,01804 |
| −24,073 | Pcdh20 | 0,00789 |
| −23,647 | Spc24 | 0,04942 |
| −23,545 | Pole4 | 0,02292 |
| −23,431 | Rftn2 | 0,00334 |
| −23,431 | Med1 | 0,00636 |
| −23,203 | Siah1b | 0,03408 |
| −23,063 | Myom1 | 0,04474 |
| −22,894 | Gldn | 0,00858 |
| −22,85 | Fam82a2 | 0,00137 |
| −22,823 | Lrsam1 | 0,00257 |
| −22,737 | Slc25a26 | 0,01523 |
| −22,196 | Whrn | 0,03241 |
| −22,121 | Cib2 | 0,02934 |
| −21,771 | Llgl2 | 0,04041 |
| −21,762 | Chrd | 0,02218 |
| −21,579 | Gap43 | 0,03692 |
| −21,301 | Insc | 0,00466 |
| −20,879 | Pom121 | 0,0455 |
| −20,236 | Cspg4 | 0,02366 |
| −20,134 | Rdh5 | 0,03387 |

(*Continued*)

| Coefficient ALR vs AKR | Gene symbol | *p*-value ALR vs AKR |
|---|---|---|
| −20,038 | Cdc42ep1 | 0,02045 |
| −19,92 | Cdk10 | 0,01486 |
| −19,744 | Cdk5 | 0,03799 |
| −19,741 | Nfkb2 | 0,04819 |
| −19,732 | BC049807 | 0,01701 |
| −19,624 | Slc6a12 | 0,04291 |
| −19,532 | Taf6 | 0,02794 |
| −19,5 | Lamc3 | 0,04393 |
| −19,224 | Txnrd2 | 0,03043 |
| −19,174 | 1810048J11 | 0,04116 |
| −18,717 | Myo1c | 0,02455 |
| −18,627 | Smoc1 | 0,01939 |
| −18,099 | Polr3d | 0,01687 |
| −17,944 | C85492 | 0,03177 |
| −17,841 | Serpina10 | 0,0084 |
| −17,677 | Pam | 0,01301 |
| −17,645 | Tmem101 | 0,03689 |
| −17,437 | Stat6 | 0,0406 |
| −17,406 | Rtn4ip1 | 0,01335 |
| −17,095 | AU040829 | 0,04191 |
| −16,874 | Pik3cb | 0,02994 |
| −16,841 | Dhx57 | 0,0498 |
| −16,833 | Col5a2 | 0,01746 |
| −16,409 | Txnrd1 | 0,01524 |
| −16,072 | Txnrd2 | 0,02834 |
| −16,069 | Zfp607 | 0,01786 |
| −15,915 | Dclk3 | 0,00929 |
| −15,722 | Tbx1 | 0,02806 |
| −15,678 | Fgf5 | 0,01348 |
| −15,166 | Abcd4 | 0,04862 |
| −14,899 | 2010007H12 | 0,01789 |
| −14,213 | Asb2 | 0,02601 |
| −14,076 | Hcn2 | 0,04652 |
| −13,992 | Timm44 | 0,04806 |
| −13,927 | 2310005N01 | 0,01499 |
| −13,912 | Fam26e | 0,04014 |
| −13,898 | Sugt1 | 0,00938 |

(*Continued*)

| Coefficient ALR vs AKR | Gene symbol | *p*-value ALR vs AKR |
|---|---|---|
| −13,896 | Ifnar2 | 0,04379 |
| −13,831 | Dgkz | 0,03023 |
| −13,181 | Psmd11 | 0,04098 |
| −13,042 | Freq | 0,0454 |
| −13,025 | Klhl30 | 0,00873 |
| −12,98 | Arid5a | 0,04258 |
| −12,916 | Phtf2 | 0,03739 |
| −12,86 | Dus2l | 0,04634 |
| −12,641 | Slc7a6os | 0,04763 |
| −12,486 | C1qtnf1 | 0,02866 |
| −12,352 | Laptm4b | 0,02804 |
| −12,217 | Kcnk2 | 0,03931 |
| −12,213 | Fam131a | 0,04824 |
| −12,193 | Slc12a4 | 0,04746 |
| −12,127 | Btbd11 | 0,03751 |
| −12,097 | Sip1 | 0,03526 |
| −12,085 | Dtna | 0,02999 |
| −12,041 | Sgcd | 0,02545 |
| −12,029 | Rasgrp3 | 0,03171 |
| −11,993 | Hoxa13 | 0,02617 |
| −11,912 | Rbm45 | 0,03592 |
| −11,897 | Hoxc13 | 0,02192 |
| −11,576 | Fam53b | 0,04918 |
| −11,542 | Adam26a | 0,03464 |
| −11,468 | Gtpbp8 | 0,04105 |
| −11,273 | Hagh | 0,04057 |
| −11,165 | Ntn3 | 0,04229 |
| −11,156 | Adssl1 | 0,03002 |
| −10,959 | Plekhb1 | 0,04069 |
| −10,827 | Ntn5 | 0,0414 |
| −10,584 | Rapgef3 | 0,03248 |
| −10,505 | Thap3 | 0,03258 |
| −10,493 | Chek2 | 0,03905 |
| −10,283 | Agpat3 | 0,04234 |
| −10,034 | Tnfrsf10b | 0,0492 |
| −9,62 | BC005624 | 0,04115 |
| −9,48 | Epha4 | 0,01966 |

(*Continued*)

| Coefficient ALR vs AKR | Gene symbol | *p*-value ALR vs AKR |
|---|---|---|
| −9,45 | Mfsd9 | 0,04124 |
| −9,085 | Cacna1h | 0,02739 |
| −8,923 | Sh3tc2 | 0,04075 |
| −8,898 | Pspc1 | 0,04049 |
| −8,853 | Tnfrsf10b | 0,03121 |
| −8,661 | Dos | 0,04554 |
| −8,572 | Pik3cd | 0,02483 |
| −8,354 | Diras1 | 0,03798 |
| −8,053 | 9630028B13 | 0,03494 |
| −7,928 | Prss36 | 0,04499 |
| −7,9 | Rhobtb2 | 0,04556 |
| −7,683 | Tars | 0,03385 |
| −7,537 | Polr3a | 0,04484 |
